# Whole-body and segmental analysis of body composition in adult males with achondroplasia using dual X-ray absorptiometry

**DOI:** 10.1371/journal.pone.0213806

**Published:** 2019-03-19

**Authors:** David Sims, Gladys Onambélé-Pearson, Adrian Burden, Carl Payton, Christopher Morse

**Affiliations:** Health, Exercise and Active Living Research, Manchester Metropolitan University, Manchester, England; Charles P. Darby Children's Research Institute, 173 Ashley Avenue, Charleston, SC 29425, USA, UNITED STATES

## Abstract

Achondroplasia is a condition characterized by a genetic mutation affecting long bone endplate development. Current data suggests that the bone mineral content (BMC) and bone mineral density (BMD) of achondroplasic populations are below age matched individuals of average stature (controls). Due to the disproportionate limb-to-torso length compared to controls however, the lower BMC and BMD may be nullified when appropriately presented. The aim of this study was to measure whole-body and segmental body composition in adult males with achondroplasia (N = 10, 22 ±3 yrs), present data relative to whole-body and whole-limb values and compare all values to age matched controls (N = 17, 22 ±2 yrs). Dual X-ray absorptiometry (DEXA) was used to measure the *in vivo* mass of the whole-body and 15 segments, from which BMD, BMC, fat free mass (FFM) and body fat mass were measured. BMC of lumbar vertebrae (L1-4) was also measured and presented as a volumetric BMD (BMD_VOL_). The achondroplasic group had less BMC, BMD and FFM, and more body fat mass than controls as a whole-body measure. The lower achondroplasic BMC and BMD was somewhat nullified when presented relative to whole-body and whole-limb values respectively. There was no difference in lumbar BMD_VOL_ between groups. Whole-body BMD measures presented the achondroplasic group as ‘osteopenic’. When relative to whole-limb measures however, achondroplasic BMD descriptions were normal. Further work is needed to create a body composition database for achondroplasic population’s, or for clinicians to present achondroplasic body composition values relative to the whole-limb.

## Introduction

Achondroplasia is the most common genetic form of dwarfism and is classically characterized by disproportionate limb-to-torso length and shorter stature (< 1.47 m) compared to persons of average stature without a form of dwarfism (hereafter referred to as ‘controls’). Achondroplasia is brought about by a fibroblast mutation resulting in shorter long-bones which presents a ‘disproportionate’ limb-to-torso lengths compared to controls [1–6]. Despite the available data on the condition, little quantitative confirmation of body composition (here defined as bone mineral content (BMC), bone mineral density (BMD), fat mass and fat free mass (FFM)) has been made. Attempts have been made to describe BMC, BMD, FFM and fat mass in achondroplasic populations but only in heterogeneous groups such as: children during maturation [7], male and female achondroplasic populations of differing ages [8–10] and, case reports [11]. The participant inclusion criteria and *in vivo* body mass evaluation methods used in these studies are therefore not robust enough to allow appropriate comparisons within achondroplasic populations or to commonly used reference data (e.g. controls).

While whole-body values of body composition are of interest to the clinician, the composition of individual segments and regions are recommended to help define clinical status instead of using whole-body measures, such as body mass index (BMI) [12]. However, providing a clinical status from whole-body measurement may be inappropriate for the achondroplasic individual. Indeed, achondroplasic groups have a higher body fat percentage than controls when assessed with skinfold calipers [7, 8], water densitometry [8] and DEXA [13]. But, due to the disproportionate limb lengths and shorter stature of achondroplasic individuals, it is therefore essential to consider the appendicular distribution of body composition rather than whole-body measures alone. For example, we have recently shown that achondroplasic adults have a lower muscle volume (here as FFM) compared to controls when using ultrasonography [13]. There appears to be, however, no conclusive whole-body or segmental body composition measures made in a homogenous achondroplasic population.

Considering achondroplasia is a genetic condition that influences the development of the long-bones, an accurate description of BMC and BMD is essential, but is underreported. Clinically, BMC and BMD are used to describe bone density, quality, strength and define osteoporosis; a systemic skeletal disease which is characterized by reduced bone tissue [14, 15]. There are empirical data from achondroplasic cohorts that suggest BMC and BMD in the femur [11, 16], spine [9] and mandible [9, 10] are lower than controls when presented as Z-scores and therefore the achondroplasic population could be at a greater risk of bone fractures. Bone health (here as BMD) is assessed using either Z or T scores but using such methods to assess bone health in achondroplasic populations is difficult due to their disproportionate limb-to-torso lengths relative to controls. The lower absolute values of BMC and BMD are likely due to the smaller achondroplasic limb length. Furthermore, studies observing BMC and BMD in achondroplasic groups have predominantly used DEXA as the mode of data collection [9–11]. All BMD measurements using DEXA are given as a ratio of BMC to area view (g·cm^-2^). Individuals with achondroplasia have irregular shaped long bones compared to controls [1, 17] which may alter the interpretation of bone density, and therefore their definition of bone health, when compared to controls. Therefore, the calculation of whole-body achondroplasic BMD is likely to be inaccurate when compared to controls when using Z and T scores. The calculation of volumetric BMD (BMD_VOL_) may be more appropriate to compare achondroplasic BMD to controls as it takes into account a greater amount of the observed bone. For example, in shorter statured groups, BMD is lower than taller groups but similar when presented as BMD_VOL_ [18, 19]. It would therefore be more useful to describe the BMD_VOL_, such as the lumbar vertebra, of achondroplasic groups to provide a more accurate representation of BMD compared to controls.

The aims of the current study were to therefore 1) measure and compare BMC, BMD, FFM and body fat between achondroplasic adults and controls, and 2) present BMC, BMD, FFM and body fat relative to appropriate anatomical measures between groups.

## Materials and method

After written informed consent, 10 adult males (22 ±3 yrs), medically confirmed as exhibiting Achondroplasia, and 17 age-matched controls (22 ±2 yrs) agreed to partake in this study. All were free from any injury for at least 3 months and self-reported good health (anthropometric descriptions of each group are given in Table 1). Ethical approval was attained from the local committee (Manchester Metropolitan University) and conformed to the latest revision of the Declaration of Helsinki. Each participant attended one testing session at the laboratories of Manchester Metropolitan University where whole-body anthropometric measurements were carried out. The study was a cross-sectional, between subject design which was developed in line with the Strengthening the Reporting of Observational Studies in Epidemiology (STROBE) statement.

### Whole-body densitometry

After fasting for ~8 hrs, a DEXA scanner (Hologic Discovery, Vertec Scientific Ltd, UK) was used to measure total mass (kg), BMC (kg), BMD (g·cm^-2^), FFM (kg) and body fat (kg) of the whole-body. Participants wore a loose-fitting cotton gown and lay supine in a predefined, anatomical position that ensured enough space was between each arm and the torso, and between each leg. The feet were positioned in an internally rotated position; participant comfort was maintained using medical tape (Transpore^TM^ Medical Tape, 3M^TM^, USA) which was wrapped around both feet to keep the leg in the required position and reduce muscle activation through the scanning protocol. A default whole-body scan (EF 8.4 lSv) was selected for all trials; scans emitted dual energy (140/100 kVp) fan-beam x-rays and lasted for ~7 minutes. The scanning region was 195 cm x 65 cm with 1.3 cm line spacing and a 0.2 cm point resolution. Each participant was exposed to ~8.4 μSv [20]. Glickman et al. [21] showed that DEXA gives a reliable measurement of whole-body mass (r = 0.940), fat (r = 0.970) and lean mass (r = 0.890) against computer tomography (CT) in controls. Similar correlations are observed in obese comparisons to CT with measures of trunk fat mass (r = 0.940), leg fat mass (r = 0.940) and leg FFM (r = 0.760) all being reliable [22]. In addition, the interrater reliability of DEXA scanning has been shown to be in excess of 0.998 [23].

### Segmental definitions

**Fig 1:**
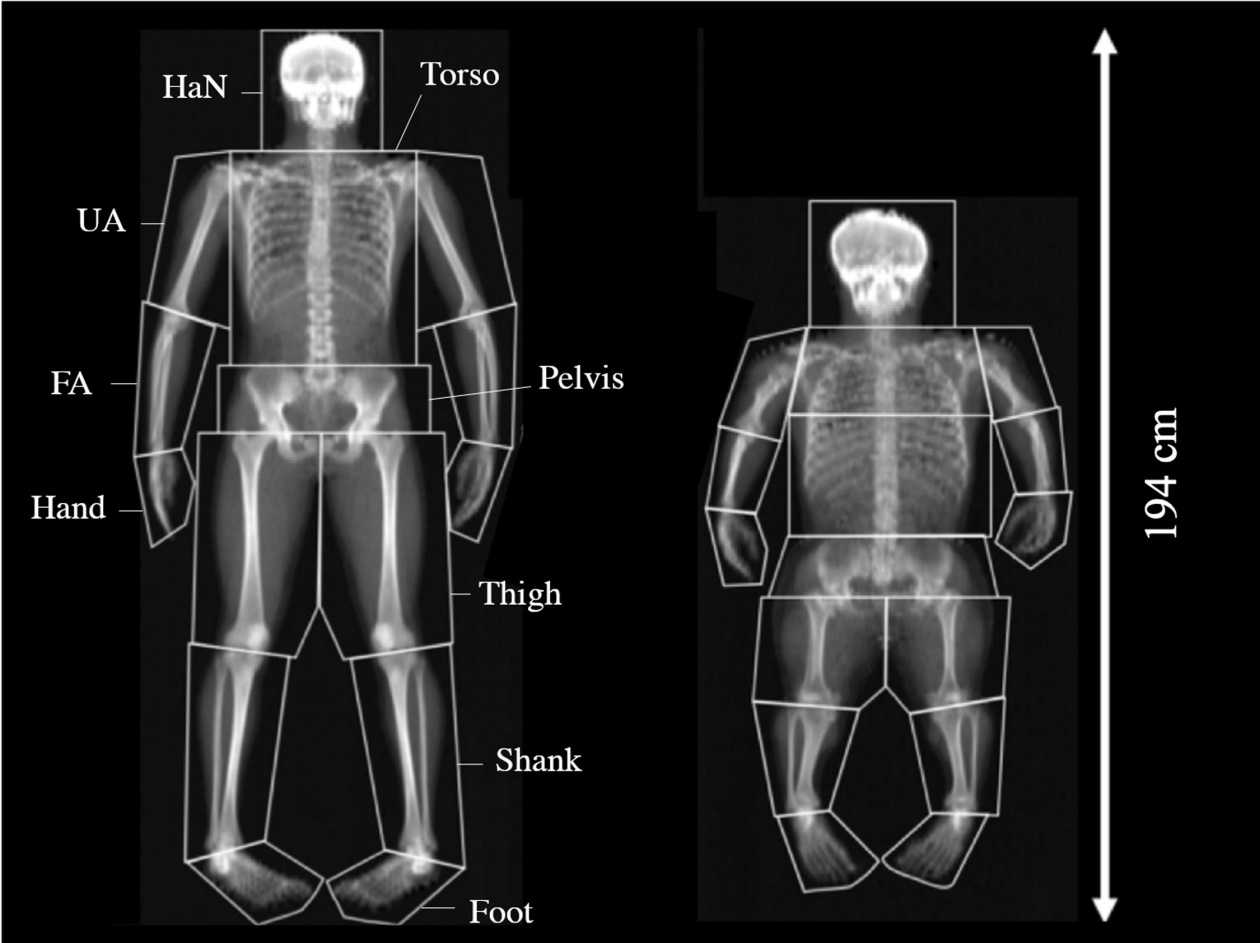
DEXA scans for (left) control and (right) achondroplasia. Segmental analysis and shown as head and neck (HaN), torso and pelvis for axial segments and upper arm (UA), forearm (FA), hand, thigh, shank and foot for the appendicular segments. Note: the thorax of achondroplasia was divided into two segments and then summed after analysis to ensure the correct mass was encompassed in the region.

Following DEXA scanning, each scan was split into 15 segments (Fig 1) using descriptions by Dempster [24]. Central segments were defined as: Head and Neck (HaN); thorax, and; pelvis. The appendicular skeleton was segmented into and defined as: upper arm (UA); forearm (FA); hand; thigh; shank, and; foot. In addition, a secondary segmentation was conducted with the HaN, thorax and pelvis combined (HTP) and the left and right limbs being summed such that whole-arm was the sum of UA, FA and hand, while the whole-leg was the sum of thigh, shank and foot. Analysis of groups’ whole-body body composition was conducted post scan with segmental analyzes conducted based on previous methods [25, 26]. Digitization of scans were completed using Physician’s View v6.1 software (Hologic, UK) with segments separated using a series of squares, rhomboids and pentagons along the transverse axis of each respective joint (Fig 1).

### Volumetric analysis of lumbar BMD

Post DEXA scan analysis was also used to segment the lumbar region of the spine (superior transverse plane of L1 to inferior transverse plane of L4, defined as L1-L4) using digitizing software (Image J, National Institute of Health, Version 1.03i) to estimate the BMC of L1-L4 (BMC_LUM_). Two of the 10 achondroplasic participants vertebral column were not identifiable post scan and so were omitted from this analysis. The lumbar vertebral column (L1-4) was assumed cylindrical and reliable methods (R = 0.979–0.992) previously described [27–29] were then used to measure the width of the lumbar vertebral column and its BMC. BMD_VOL_ was then calculated as:

$${\text{Lum}}_{\text{VOL}}\;\text{=}\;\pi \cdot {\;\text{r}}^{\text{2}}\;\cdot \;\text{H}$$

$${\text{BMD}}_{\text{VOL}}\;\text{=}\;\frac{{\text{BMC}}_{\text{LUM}}}{{\text{Lum}}_{\text{VOL}}}$$

**Fig 2:**
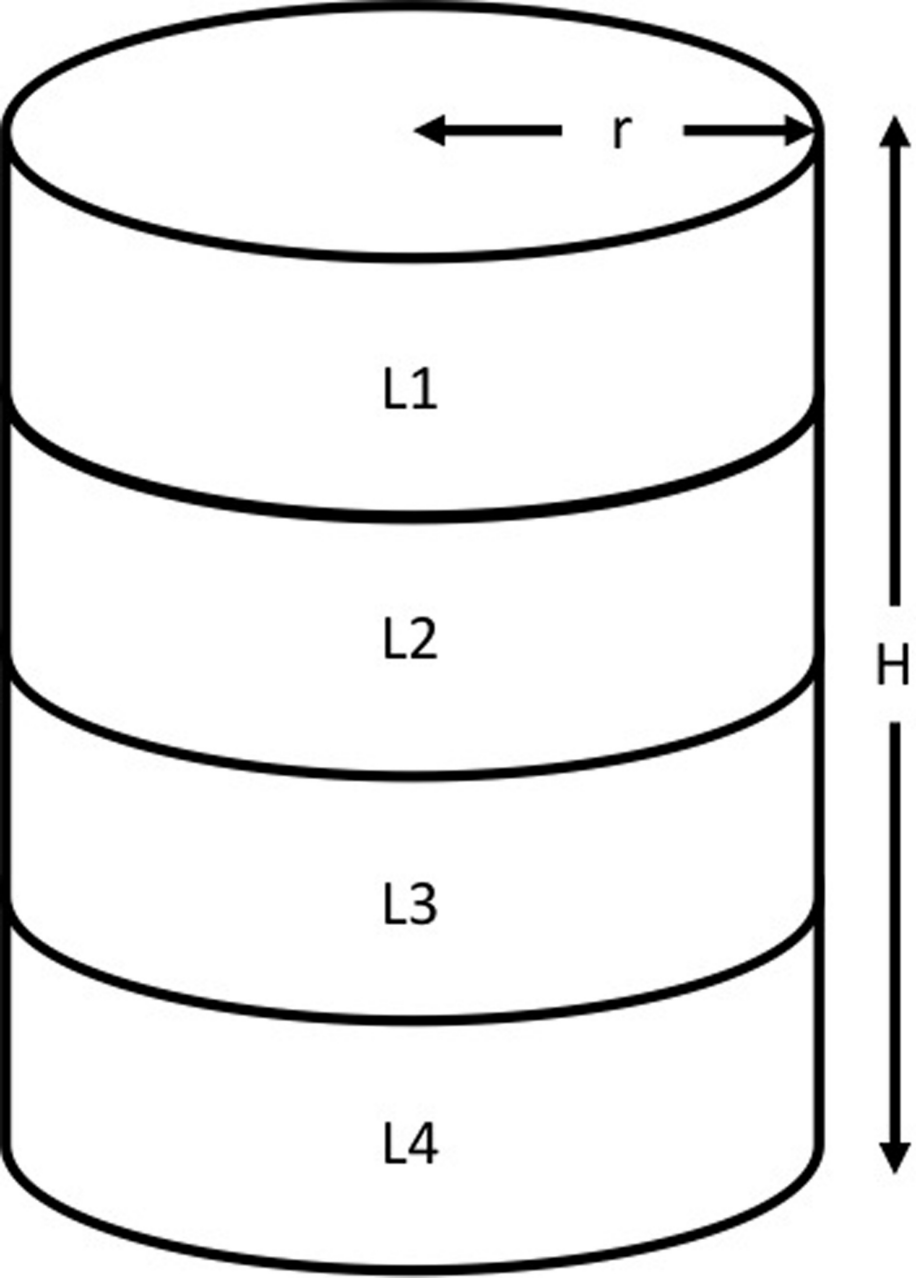
Volumetric calculations of BMD using DEXA data. Where r is radius, H is the height of the lumbar column, L1-4 is each of the respective lumbar vertebrae, adapted from Kröger et al.[34].

Where Lum_VOL_ is the volume of the lumbar (L1-L4) vertebrae, π is 3.14, r is the radius of the lumbar (i.e. half the width measured by ImageJ), H is lumbar column’s height measured by ImageJ, BMC_LUM_ is the bone mineral content of the lumbar region measured by DEXA and BMD_VOL_ is the volumetric bone mineral density of the lumbar column (Fig 2).

### Statistical analysis

All body composition variables were initially compared between groups as a whole-body measure. Following, each individual segment was presented relative to whole-body values (%) and relative to its respective limb (%) as described in the segmental definitions section of this study’s method method. All data were collated on a personal computer (Macintosh, California) and inferentially analyzed using SPSS (v22.0, IBM). A multivariate analysis of variance (MANOVA) was used to establish differences between left and right sides and between groups. Independent t-tests were carried out on the whole-body, central segments and BMD_VOL_ values between groups. For variables that violated parametric assumptions, a Mann-Whitney U were performed to assess between-group differences of the central segments, while data that violated Levene’s test were corrected using the non-equal variance option in SPSS (Greenhouse Geisser). Alpha was set at < 0.05 with all results reported as means (SD).

## Results

### Whole-body measures

There was no difference in age between groups (P = 0.487, Table 1). The achondroplasic group were 23% smaller in stature (P < 0.001), had 19% less body mass (P < 0.001) and had a 25% greater BMI (P < 0.001) than controls (Table 1). As whole-body measure, the achondroplasic group had 15% less BMD (P < 0.001), 32% less BMC (P < 0.001) and 26% less FFM (P < 0.001) than controls (Table 1). There was no difference in fat mass between groups (P = 0.447), but the achondroplasic group had a higher body fat percentage than controls (P < 0.001, Table 1).

**Table 1:** Anthropometric data for the achondroplasic and control groups, values displayed as mean (SD).

	Achondroplasia (N = 10)		Control (N = 17)
Stature (m) ^†^	1.38 (0.05)	^‡^	1.79 (0.08)
Total mass (kg)	61.9 (8.7)	^‡^	76.5 (10.6)
Body Mass Index (kg·m^-2^)	32.4 (3)	^‡^	24.1 (4.5)
BMC (kg)	2.1 (0.3)	^‡^	3.1 (0.5)
BMD (g·cm^-2^)	1.17 (0.10)	^‡^	1.37 (0.11)
FFM (kg)	41.3 (5.3)	^‡^	55.6 (7.6)
Body Fat (kg)	18.3 (3.9)		16.8 (5.0)
Body Fat (%)	29.3 (2.9)	^‡^	22.4 (5.3)
BMC_LUM_ (g)	62.5 (13.8)	^‡^	90.4 (15.3)
BMD_VOL_ (g·cm^-3^) ^†^	0.290 (0.051)		0.279 (0.044)

BMC, Bone Mineral Content; BMD, Bone Mineral Density; FFM, Fat Free Mass; BMC_LUM_, Bone Mineral Content of the Lumbar Vertebrae (L1-4);

^†^ Mann Whitney-U t-test.

^‡^ P ≤ 0.001.

### Segment analysis

MANOVA showed no difference in any body composition measure between left and right limbs. Therefore, data are presented as mean values of the left and right segments and limbs. The total mass of the all achondroplasic segments was lower than controls (P < 0.001) and there were significant effects between groups’ BMC, BMD, FFM and body fat mass (P < 0.001, Table 2).

**Table 2:** Total segment mass, BMC, BMD, FFM and body fat for the achondroplasic group and controls. Values displayed as mean (SD).

	BMC (kg)			BMD (g·cm^-2^)			FFM (kg)			Body Fat (kg)	
	Achon		Control	Achon		Control	Achon		Control	Achon	Control
HaN	0.62 (0.08)		0.59 (0.08)	2.11 (0.21)		2.17 (0.25)	3.92 (0.49)		3.73 (0.28)	1.33 (0.16)	1.24 (0.09)
Thorax	0.42 (0.11)		0.47 (0.07)	1.73 (1.02)		1.32 (0.59)	16.39 (1.89)	^†^	19.43 (2.48)	6.18 (1.80)	5.09 (1.89)
Pelvis	0.20 (0.06)	^†^	0.28 (0.06)	1.15 (0.37)	^‡^	1.37 (0.18)	5.34 (0.89)		6.05 (1.32)	2.33 (0.51)	1.91 (0.60)
UA Right	0.05 (0.01)	^*^	0.10 (0.02)	1.00 (0.09)	^*^	1.19 (0.14)	0.86 (0.20)	^*^	1.83 (0.31)	0.57 (0.14)	0.58 (0.22)
FA Right	0.05 (0.01)	^*^	0.08 (0.02)	0.94 (0.14)	^†^	1.07 (0.15)	0.58 (0.09)	^*^	0.96 (0.13)	0.23 (0.06)	0.21 (0.08)
Hand	0.02 (0.01)	^†^	0.03 (0.01)	0.54 (0.09)	^†^	0.64 (0.13)	0.22 (0.04)	^*^	0.31 (0.05)	0.12 (0.02)	0.10 (0.05)
Thigh	0.13 (0.04)	^*^	0.36 (0.07)	1.18 (0.11)	^*^	1.72 (0.19)	4.45 (0.84)	^*^	7.50 (1.55)	2.35 (0.49)	2.46 (0.84)
Shank	0.12 (0.02)	^*^	0.24 (0.04)	1.04 (0.08)	^*^	1.37 (0.18)	1.25 (0.16)	^*^	2.19 (0.32)	0.74 (0.19)	0.77 (0.30)
Foot	0.04 (0.01)	^*^	0.07 (0.02)	0.79 (0.09)	^*^	1.11 (0.21)	0.46 (0.05)	^*^	0.64 (0.09)	0.20 (0.04)	0.18 (0.06)
HTT	1.24 (0.20)		1.34 (0.16)	4.99 (1.46)		4.86 (0.75)	25.64 (3.13)	^‡^	29.21 (3.49)	9.83 (9.83)	8.25 (2.46)
Whole Arm	0.12 (0.02)	^*^	0.21 (0.04)	2.47 (0.28)	^*^	2.90 (0.35)	1.66 (0.31)	^*^	3.11 (0.45)	0.92 (0.19)	0.89 (0.32)
Whole Leg	0.29 (0.05)	^*^	0.68 (0.13)	3.00 (0.24)	^*^	4.21 (0.46)	6.15 (0.96)	^*^	10.33 (1.73)	3.29 (0.69)	3.41 (1.11)

Values displayed mean (SD). Achon, achondroplasia; HaN, Head and Neck; UA, Upper Arm; FA, Fore Arm; HTP, HaN, Torso and Pelvis;

‡ P ≤ 0.05,

† P ≤ 0.01,

* P ≤ 0.001.

### Bone mineral content

There was no difference in BMC of the HaN (P = 0.415), thorax (P = 0.234) or HTP (P = 0.166) between groups. The achondroplasic group had less BMC than controls in the pelvis (P = 0.004), UA (P < 0.001), FA (P < 0.001), hand (P = 0.001), arm (P < 0.001), thigh (P < 0.001), shank (P < 0.001), foot (P < 0.001) and leg (P < 0.001, Table 2).

BMC relative to whole-body BMC was higher in the achondroplasic group’s HaN (P < 0.001), thorax (P < 0.001), HTP (P < 0.001) and hand compared to controls (P = 0.043). Relative to whole-body BMC, pelvis BMC was not different between groups (P = 0.342, Table 3). BMC relative to whole-body BMC was lower in achondroplasic group’s UA (P < 0.001), FA (P < 0.001) whole arm (P < 0.001), thigh (P < 0.001), shank (P < 0.001), foot (P < 0.001) and whole leg compared to controls (P < 0.001, Table 3).

**Table 3:** Segmental BMC, BMD, FFM and body fat presented as a percentage (%) of each respective whole-body composition for the achondroplasic group and controls. Values displayed mean (SD).

	BMC (%)			BMD (%)			FFM (%)			Body Fat (%)		
	Achon		Control	Achon		Control	Achon		Control	Achon		Control
HaN	30.4 (2.8)	^*^	19.3 (3.3)	13.3 (1.0)	^†^	11.5 (1.7)	9.5 (0.5)	^*^	6.7 (0.9)	7.4 (1.2)		7.9 (2)
Thorax	20.3 (2.9)	^*^	15.1 (1.2)	10.5 (4.7)	^‡^	6.7 (2.5)	39.8 (1.7)	^*^	34.8 (3.0)	33.5 (4.0)		30.0 (4.6)
Pelvis	9.5 (2.3)		8.9 (1.1)	7.1 (1.5)		7.2 (0.7)	12.9 (1.2)	^*^	10.7 (1.6)	12.8 (1.6)	^‡^	11.3 (1.2)
UA	2.4 (0.3)	^*^	3.2 (0.3)	6.3 (0.6)		6.2 (0.4)	2.1 (0.3)	^*^	3.3 (0.4)	3.1 (0.4)	^‡^	3.4 (0.4)
FA	2.4 (0.3)	^‡^	2.6 (0.5)	5.9 (0.8)		5.6 (0.7)	1.4 (0.1)	^*^	1.7 (0.2)	1.3 (0.2)		1.2 (0.2)
Hand	1.1 (0.3)	^‡^	1.0 (0.2)	3.4 (0.6)		3.4 (0.6)	0.5 (0.1)		0.6 (0.1)	0.7 (0.1)		0.6 (0.2)
Thigh	6.4 (1.8)	^*^	11.5 (1.1)	7.4 (0.8)	^*^	9.0 (0.5)	10.8 (1.6)	^*^	13.3 (1.6)	12.9 (1.1)	^†^	14.6 (2.2)
Shank	5.7 (0.6)	^*^	7.7 (0.6)	6.5 (0.6)	^†^	7.2 (0.9)	3.0 (0.3)	^*^	3.9 (0.6)	4.1 (0.6)	^‡^	4.5 (0.8)
Foot	1.9 (0.3)	^*^	2.4 (0.4)	5.0 (0.6)	^†^	5.8 (0.9)	1.1 (0.1)		1.2 (0.2)	1.1 (0.2)		1.1 (0.3)
HTP	60.2 (3.1)	^*^	43.2 (3.5)	30.9 (5.4)	^‡^	25.4 (2.3)	62.2 (2.5)	^*^	52.2 (2.2)	53.7 (3.0)	^‡^	49.2 (5.0)
Whole Arm	5.9 (0.7)	^*^	6.9 (0.6)	15.6 (1.7)		15.2 (1.1)	4.0 (0.4)	^*^	5.6 (0.5)	5.1 (0.5)		5.2 (0.6)
Whole Leg	14 (1.9)	^*^	21.5 (1.8)	18.9 (1.7)	^*^	22.1 (1.6)	14.9 (1.7)	^*^	18.3 (1.2)	18.1 (1.6)	^*^	20.2 (2.3)

Values displayed mean (SD). Achon, achondroplasia; HaN, Head and Neck; UA, Upper Arm; FA, Fore Arm; HTP, HaN, Torso and Pelvis;

‡ P ≤ 0.05,

† P ≤ 0.01,

* P ≤ 0.001.

BMC relative to HTP was not different between groups in the thorax (P = 0.245) but was higher in the achondroplasic group’s HaN and pelvis than controls (P = 0.004 and P = 0.002 respectively, Table 4). The achondroplasic group’s BMC of the UA and FA, relative to whole-arm BMC, was lower than controls (P < 0.001 and P = 0.023), but higher in the hand than controls (P < 0.001, Table 4). Relative to whole-leg BMC, the achondroplasic group had a lower thigh BMC (P < 0.001) but a higher shank and foot BMC than controls (P < 0.001 and P < 0.001 respectively, Table 4).

**Table 4:** Segmental BMC, BMD, FFM and body fat presented as a percentage (%) of each segment’s respective whole-limb body composition mass for the achondroplasic group and controls. Values displayed mean (SD).

	BMC (%)			BMD (%)			FFM (%)			Body Fat (%)		
	Achon		Control	Achon		Control	Achon		Control	Achon		Control
HaN	50.6 (5.2)	^‡^	44.4 (4.8)	44.1 (7.3)		45.4 (7.1)	15.3 (0.9)	^*^	12.9 (1.5)	13.9 (2.5)		16.1 (4.0)
Thorax	33.6 (3.4)		34.9 (2.6)	32.8 (8.0)	^‡^	26.1 (7.6)	64.0 (1.5)	^‡^	66.5 (3.2)	62.2 (5.0)		60.7 (4.4)
Pelvis	15.8 (3.6)	^‡^	20.6 (3.3)	23.1 (3.4)	^*^	28.5 (3.3)	20.7 (1.4)		20.6 (3.3)	23.9 (3.2)		23.1 (2.3)
UA	41.2 (3.5)	^*^	47.5 (5.3)	40.5 (2.9)		41.2 (3.4)	51.7 (3.3)	^*^	58.8 (2.9)	61.7 (3.3)	^†^	64.9 (3.7)
FA	39.8 (2.3)		38.2 (6.3)	37.8 (1.7)		36.8 (2.6)	35.1 (2.9)	^*^	31.1 (1.9)	25.3 (2.8)	^‡^	23.4 (2.6)
Hand	19.0 (3.1)	^*^	14.3 (3.4)	21.7 (2.1)		22.0 (2.8)	13.3 (1.5)	^*^	10.1 (1.5)	13.0 (1.9)		11.7 (2.7)
Thigh	44.6 (1.4)	^*^	53.4 (1.9)	39.2 (1.4)	^†^	41.0 (2.3)	71.8 (4.2)		72.0 (5.8)	71.6 (2.7)		72.0 (5.8)
Shank	41.5 (7.3)	^*^	35.7 (1.7)	34.6 (1.4)	^‡^	32.7 (2.9)	20.6 (3.0)		21.6 (4.5)	22.4 (2.4)		22.7 (6.1)
Foot	14.0 (3.0)	^*^	10.9 (1.3)	26.2 (1.6)		26.3 (2.8)	7.5 (1.4)	^†^	6.4 (1.5)	6.0 (0.7)		5.3 (1.4)

Values displayed mean (SD). Achon, achondroplasia; HaN, Head and Neck; UA, Upper Arm; FA, Fore Arm; HTP, HaN, Torso and Pelvis;

‡ P ≤ 0.05,

† P ≤ 0.01,

* P ≤ 0.001.

### Bone mineral density

There was no difference in HaN or thorax BMD between groups (P = 0.526 and P = 0.190, respectively). The achondroplasic group had lower BMD of the UA (P < 0.001), FA (P = 0.004), hand (P = 0.002), whole-arm (P < 0.001), pelvis (P = 0.047), thigh (P < 0.001), shank (P < 0.001), foot (P < 0.001) and whole-leg compared to controls (P < 0.001, Table 2). There was no difference in BMD of HTP between groups (P = 0.546).

Relative to whole-body BMD, the achondroplasic group had a higher BMD of the HaN (P = 0.004), thorax (P = 0.011) and HTP (P < 0.001) than controls, but no differences were observed between groups’ pelvis (P = 0.822), UA (P = 0.634), FA (P = 0.141), hand (P = 0.812) or whole-arm (P = 0.432, Table 3). Relative to whole-body BMD, the achondroplasic group had a lower BMD of the thigh (P < 0.001), shank (P = 0.005), foot (P = 0.001) and whole-leg compared to controls (P < 0.001, Table 3).

Relative to HTP BMD, there was no difference in thorax BMD between groups (P = 0.637), but the achondroplasic group had a higher HaN (P = 0.039) and a lower pelvis BMD than controls (P < 0.001, Table 4). Relative to whole-arm BMD, there was no difference in UA (P = 0.485), FA (P = 0.155) or hand (P = 0.668) BMD between groups (Table 4). Relative to whole-leg BMD, the achondroplasic group had a lower thigh (P = 0.002), but a higher shank BMD than controls (P = 0.011, Table 4). No difference in foot BMD relative to whole-leg BMD existed between groups (P = 0.857, Table 4).

### Lumbar measures

The achondroplasic group had 31% less BMC_LUM_ compared to controls (P = 0.001). There was no group difference in BMD_VOL_ (P = 0.597, Table 1).

### Fat free mass

There was no difference in FFM between the achondroplasic groups and controls for the HaN or pelvis (P = 0.217 and P = 0.365, respectively), but the achondroplasic groups had less thorax and HTP FFM than controls (P = 0.005 and P = 0.013 respectively, Table 2). The achondroplasic group has less UA (P < 0.001), FA (P < 0.001), hand (P < 0.001) and whole-arm FFM than controls (P < 0.001, Table 2). The achondroplasic group also had less thigh (P < 0.001), shank (P < 0.001), foot (P < 0.001) and whole-leg FFM than controls (P < 0.001, Table 2).

Relative to whole-body FFM, the achondroplasic group had more HaN (P < 0.001), thorax (P < 0.001), pelvis (P < 0.001) and HTP FFM than controls (P < 0.001, Table 3). The achondroplasic group has less FFM, relative to whole-body FFM, than controls in all other segments (P < 0.05) other than hand and foot when no differences were observed (P = 0.125 and P = 0.022, respectively, Table 3).

Relative to HTP FFM, the achondroplasic group had more HaN and less thorax FFM than controls (P < 0.001 and P = 0.027, respectively); no difference in pelvis FFM between groups existed (P = 0.894). Relative to whole-arm FFM, the achondroplasic group had less UA and hand FFM (P < 0.001 and P < 0.001, respectively), but more FA FFM compared to controls (P < 0.001, Table 4). Relative to whole-leg FFM, there was no difference thigh or shank FFM between groups (P = 0.910 and P = 0.388, respectively), but the achondroplasic group had more foot FFM than controls (P = 0.008, Table 4).

### Body fat

There was no difference in the amount of body fat mass between groups in any segment (P > 0.05, Table 2).

When relative to whole-body fat mass, there was no difference between groups’ HaN or thorax fat (P = 0.524 and P = 0.061, respectively), but the achondroplasic group had more pelvis and HTP fat compared to controls (P = 0.013 and P = 0.017, respectively, Table 3). There was no difference in FA (P = 0 .299), hand (P = 0.323) or whole arm fat (P = 0.431) when relative to whole-body fat, but the achondroplasic group had more UA fat than controls (P = 0.025, Table 3). When relative to whole-body fat, the achondroplasic group had less thigh (P = 0.003), shank (P = 0.034) and whole-leg fat than controls (P = 0.001); there was no difference in groups’ foot fat (P = 0.865, Table 3).

There were no differences in groups’ HaN, thorax or pelvis fat when relative to HTP fat (P > 0.05, Table 4). Relative to whole-arm fat, the achondroplasic group had less UA and FA fat compared to controls (P = 0.003 and P = 0.013, respectively); no difference in hand fat was observed (P = 0.066, Table 4). There was no difference in thigh (P = 0.770), shank (P = 0.847) or foot fat between groups when relative to whole-leg fat (P = 0.053, Table 4).

## Discussion

The aims of this study were to assess the whole-body and segment *in vivo* BMC, BMD, FFM and fat mass of achondroplasic adults, present values relative to respective whole-body and whole-limb values and compare all values to controls. The findings show that adult achondroplasic males have less BMC, BMD and FFM than controls at the whole-body and segmental level, but body fat masses are the same as controls. The differences in segmental body composition are nullified somewhat when relative to whole-body and whole-limb measures.

### Bone mineral content and density

Bone density is associated with fracture risk in all populations [30]. As shown here, the achondroplasic group had a lower BMC and BMD of the whole-body and individual body segments. Similar results have been observed at the whole-body level [10, 11] and in the achondroplasic mandible and lumbar spine [9]. Our results are only comparable to a few participants included in those studies due to the participant demographics and dysplasic classifications used in both studies. In the current study, the lower absolute BMC of the achondroplasic appendicular segments suggest that the mutated FGFR3 gene not only results in shorter ‘long’ bones but may impact BMC within the bones. The lower BMD in the achondroplasic group are unsurprising given that the mutated FGFR3 gene results in shorter bones [31] and therefore less viewable area when DEXA scanning resulting in an under prediction of long bone’s BMD [32]. This may be the case in the current study as whole-body and segmental values of BMC and BMD were lower than controls, to the point where the achondroplasic group had a whole-body BMD Z-score of -1.82. This would classify the achondroplasic group with osteopenia and at a ‘higher risk’ of bone fractures using the controls as reference data [33]. In the current study, we made BMC and BMD relative to whole-body and whole-limb masses to allow for a more informed comparison between groups. The relative presentations of BMC and BMD did appear to nullify the osteopenic classification of the achondroplasic group. The use of whole-body BMD to compare bone quality between groups of different limb length proportions, such as Achondroplasia, may therefore lead to a misinterpretation of clinical state.

The BMD_VOL_ was similar between groups despite the lower BMC of the achondroplasic group. Similar results of BMD_VOL_ are observed in the literature when different sized vertebra are compared [18, 34]. When relative to whole-body values, the differences in groups’ BMC and BMD remained, but when relative to whole-limb values BMC and BMD differences appeared to nullify and, at times, reverse. For example, achondroplasic segmental BMC was lower than controls when relative to whole-body BMC, suggesting that the mutated FGFR3 gene indeed alters achondroplasic bone structure and quality rather than just the end plates [35]. However, when the shank and foot BMC were presented relative to whole-leg BMC, the achondroplasic group had a higher BMC than controls. Similarly, achondroplasic BMD of the shank and foot, when relative to whole-leg values, were higher than controls which suggest achondroplasic bone health is greater than controls for the bones in these segments. It is possible that these results are due to a higher magnitude of force and a more frequent application of force during activities of daily living (e.g. walking) for the achondroplasic group than controls. This can partially be explained by the presented results as the achondroplasic group have a greater upper body mass relative to whole-body mass than controls. It is therefore likely that the relative achondroplasic reaction force (ground reaction force ÷ body weight) experienced by the relatively smaller achondroplasic foot and shank when walking is larger than controls. Such results are observed elsewhere in populations where mass distribution and reaction forces are manipulated [36, 37]. Furthermore, with the achondroplasic group having shorter legs than controls [38], they are likely to have a higher stride frequency at habitual walking speeds, like that observed between groups of different stature [39, 40]. The higher relative ground reaction force and greater loading frequency would likely increase achondroplasic bone turnover, leading to a higher BMC and BMD of the lower limb segments. To back this theory though, either an *in vitro* analysis of achondroplasic lower limb bones is required, or, a longitudinal analysis combining force development during activities, such as walking and/or running, and the monitoring of BMC and BMD are required.

### Fat free mass and body fat

The lower whole-body and whole-limb FFM of the achondroplasic groups seen here would be explained, in part, by their shorter limbs and their higher whole-limb fat mass. Similar to the bone density measures, when the FFM and fat of individual segments were presented relative to whole-limb values, differences in both FFM and fat were somewhat removed; only lower FFM and fat of the achondroplasic arm segments were apparent. Despite the similar FFM in ambulatory segments, we have previously observed that the specific force development (relative force development of fascicles) of the achondroplasic vastus lateralis is lower than controls [13]. The composition of the leg segments’ FFM may therefore include more non-contractile properties (e.g. fat infiltration) which leads to an inflated achondroplasic FFM relative to whole-limb mass.

In the present study, the achondroplasic group’s BMI and body fat percentage would class them as ‘moderately obese’ and would place them ‘at risk’ of cardiovascular disease [41–45]. Higher levels of abdominal fat are associated with cardiovascular deaths [46, 47]. Previous work by Hecht et al. [48], and more recently Wynn et al. [49], report high rates of achondroplasic deaths attributed to cardiovascular disease (~32%), which could be attributed to the higher thorax and pelvis fat. While the achondroplasic group have the same fat mass as controls in the thorax and pelvis, their masses relative to whole-body values suggest a higher abdominal fat than controls and therefore a possible reason for the attributed cardiovascular deaths observed in the population. However, the achondroplasic group had less HTP fat when presented relative to whole-body fat, which contradicts the speculation above. To make any more substantial conclusions on this topic is difficult from the available data sets. Further work investigating the longitudinal analyses of achondroplasic body fat, its distribution, diet and their lifestyle (e.g. physical activity) is required to suggest correlative links to the observed higher rates of cardiovascular deaths in this population.

### Clinical implications

As a whole-body measure, the current data suggest that the achondroplasic group are at risk of a number of health complications (e.g. osteopenia and increased risk of cardiovascular events); the relative data however, suggest that this may not be the case. The commonly used normative data sets to define health states of control populations are valid given the similarity in the populations used to provide such data [50]. However, comparisons are only valid when they are done between populations of similar age, sex and stature, of which there are amply data for controls. We have demonstrated in the present study that due to the disproportionate torso-to-limb length (relative to controls) of the achondroplasic populations, such normative data sets are redundant for this population. To the authors’ knowledge, there remains no extensive body composition databases for specific achondroplasic comparisons. Therefore, the data and analysis from the current study suggests that the classification of achondroplasic health states by clinicians be made with caution, or with an attempt to present the data, as we have done here.

The achondroplasic condition is irreversible, but the development of bone following surgical procedures of bones, such as leg lengthening, appear normal [51, 52]. Certainly, from the presented BMC and BMD data of the shank and foot relative to the whole-leg values, it would be assumed that bone turnover and its development is similar to controls. It can also be assumed that increased BMC of all bones could be achieved in the achondroplasic population through appropriate interventions, as observed in ‘osteopenic’ groups. For example, in the elderly, BMD of the femoral neck [53, 54] and lumbar column [54] increase following resistance and aerobic exercise interventions, respectively. It is likely that BMC and BMD of the achondroplasic bones would improve (i.e. become higher) through similar exercise interventions. However, to date, there appears to be no structured exercise intervention aimed at improving bone health in any achondroplasic population. With the condition affecting bone end plate development and structure, it is likely that the ability of an achondroplasic person to perform complex resistance exercises is different to controls. Therefore, it would be advised that movement analyses of different exercises be explored in the achondroplasic populations prior to intervening with previously utilized exercise modes.

### Limitations

While we present and describe *in vivo* body composition of an adult male achondroplasic population, we do so in a relatively small population. The demographics of our population (i.e. aged between 18–35, male and currently recreationally physically active) represent ~14% of the U.K. population. We cannot state with confidence however, that the observed whole-body results and methods used to present relative body composition values are appropriate to other achondroplasic populations. For example, comparisons of body composition, particular BMC and BMD, are different between ages and in controls [50] and are likely to be similar in different achondroplasic populations. Indeed, had the authors had the availability of a lager achondroplasic cohort, we would had attempted to collate population specific (i.e. gender, age etc.) whole-body and segmental body composition. This study should therefore be used as an example of presenting body composition for achondroplasic populations and be the basis of a normative data set for the population as a whole.

The methods we used to measure body composition (DEXA), and the methods of segmenting the DEXA scans have been shown to be reliable [21, 25, 26]. There are certainly other methods available to conduct such analyses of body composition, for example CT or magnetic resonance imaging (MRI). While DEXA scanning has been shown to reliable assess body composition in adults with vastly different body morphologies [22], there are numerous examples in the literature that show DEXA continuously under predicts fat mass of the whole-body [55], abdominal fat [21, 22, 56] and thigh fat [22] and overpredicts BMD [57] compared to finite element analysis, such as CT or MRI. In addition, it appears that DEXA’s under prediction of fat mass compared to CT becomes greater within increasing weight [22]. It is therefore likely that both the achondroplasic and control group’s body fat are under predicted in the current study. Importantly though, were CT or MRI used instead of DEXA, the direction of the difference observed between the two groups’ fat mass are likely to be the same and the magnitude of the difference in whole-body and segmental fat and BMD between the groups are likely to be larger. Unfortunately, this study did not have access to such specialized equipment, but its data and its limitations do provide strong rationale for appropriate analyses to be used in achondroplasic groups.

## Conclusion

The aim of this study was to measure and compare body composition between achondroplasic adults and controls. The main findings of this study were that whole-body composition of the achondroplasic group classify them ‘at risk’ of a number of health complications, such as osteoporosis. Presenting achondroplasic body composition relative to whole-body and whole-limb values however, nullifies these classifications and may give a better representation of the achondroplasic groups mass distribution when compared to controls. To make valid clinical descriptions of achondroplasic populations, further work is required to either create separate body composition databases, or, for their body composition to be presented as we have done here.

## Supporting information

S1 Table Participant values of scanned area (cm^2^) for each segment. (PDF)

S2 Table Participant values of bone mineral content (kg) for each segment. (PDF)

S3 Table Participant values of bone mineral content (g·cm^-2^) for each segment. (PDF)

S4 Table Participant values of scanned fat (kg) for each segment. (PDF)

S5 Table Participant values of scanned lean mass and bone mineral content (kg) for each segment. (PDF)

S6 Table Participant values of total mass (kg) for each segment. (PDF)

S7 Table Participant values of fat (%) for each segment. (PDF)

S8 Table Participant values of scanned lean mass (kg) for each segment. (PDF)
