# Supplementary material for: Whole-body and segmental analysis of body composition in adult males with achondroplasia using dual X-ray absorptiometry
Source: PLoS One. 2019 Mar 19;14(3):e0213806. doi: 10.1371/journal.pone.0213806 (PMC6424418; doi:10.1371/journal.pone.0213806)
Supplement: S1 Table — (PDF) [file pone.0213806.s001.pdf]

S1 Table: Participant values of scanned area (cm^2) for each segment.

Participant Number	Head & Neck	Trunk		Right Arm			Left Arm			Right Leg			Left Leg		
		Thorax	Pelvis	Upper Arm	Fore Arm	Hand	Upper Arm	Fore Arm	Hand	Thigh	Shank	Foot	Thigh	Shank	Foot
Control 1	275.93	491.07	332.55	74.61	86.45	38.29	80.14	76.19	42.24	190.67	180.80	62.77	196.16	170.93	55.66
Control 2	279.09	459.89	167.77	82.11	72.24	50.13	83.69	82.90	53.29	173.69	173.30	69.87	175.67	164.61	66.32
Control 3	265.67	409.78	216.72	71.85	82.50	50.53	74.61	82.11	45.40	189.48	170.53	54.48	185.14	161.06	54.08
Control 4	245.14	419.63	140.93	55.66	57.63	40.68	58.82	56.06	41.84	147.24	125.53	44.21	146.06	116.85	42.24
Control 5	275.93	463.45	196.98	85.66	84.48	50.13	91.58	82.50	50.92	230.14	185.14	68.29	212.77	177.25	73.03
Control 6	297.65	482.39	234.49	79.35	62.77	45.40	87.64	61.58	42.63	213.17	179.61	58.82	198.17	158.30	63.95
Control 7	270.80	484.76	238.93	96.72	93.16	56.06	97.90	93.56	50.53	230.93	194.61	69.48	217.51	189.09	58.03
Control 8	288.17	485.55	249.09	80.14	73.82	47.77	75.79	86.85	48.95	223.43	184.35	68.29	224.22	171.72	63.95
Control 9	271.59	478.44	217.91	86.06	87.64	48.95	79.74	92.77	57.24	209.62	176.46	68.69	200.93	157.90	69.08
Control 10	258.57	462.63	183.17	91.19	71.85	47.77	90.40	73.82	45.40	222.64	187.51	81.71	215.14	181.19	89.61
Control 11	266.46	513.58	264.49	94.74	82.90	45.40	92.37	86.85	49.74	251.06	198.17	8.03	235.06	197.77	80.92
Control 12	293.70	552.66	211.19	92.37	88.03	51.32	99.08	98.69	56.84	207.64	191.46	72.24	204.48	198.56	68.69
Control 13	267.64	481.21	238.04	95.14	86.45	52.50	93.95	80.92	51.71	213.56	195.40	38.29	207.64	183.17	80.53
Control 14	301.20	500.16	238.43	106.58	106.58	73.03	99.08	104.22	66.32	240.01	203.69	78.56	245.93	211.98	65.92
Control 15	267.64	525.03	204.09	89.21	66.32	42.63	84.08	65.53	43.82	189.48	146.45	65.53	199.75	149.22	52.11
Control 16	243.17	548.71	207.64	88.43	87.24	48.95	92.37	75.40	53.29	220.67	181.19	79.74	202.12	175.67	75.74
Control 17	274.75	549.50	200.14	89.61	69.48	43.42	85.66	74.21	56.84	231.72	196.59	60.79	226.98	201.33	6.06
Achondroplasia 1	293.70	505.68	157.11	56.84	50.53	49.74	61.19	49.34	40.27	139.35	121.98	55.66	124.35	124.35	64.35
Achondroplasia 2	277.12	483.58	143.69	48.55	44.80	40.27	44.61	51.71	42.24	122.77	103.43	44.61	110.14	104.22	40.27
Achondroplasia 3	268.04	376.20	195.80	47.37	46.58	32.76	43.42	46.98	33.55	127.90	103.43	39.87	118.82	99.08	43.82
Achondroplasia 4	262.22	430.43	160.07	44.95	41.60	35.25	42.16	46.47	37.48	106.99	101.79	44.81	101.79	98.94	44.32
Achondroplasia 5	313.83	500.55	198.96	43.82	54.87	33.95	39.48	48.55	46.58	103.82	106.19	44.61	83.29	89.21	45.79
Achondroplasia 6	307.91	459.89	218.69	46.19	58.42	46.19	39.87	65.92	46.58	118.43	123.56	60.60	118.82	114.48	57.24
Achondroplasia 7	270.80	467.39	167.77	50.92	46.98	40.27	48.95	48.55	44.21	123.16	116.06	52.11	111.32	111.32	46.58
Achondroplasia 8	256.59	414.10	138.16	46.19	45.79	35.13	41.45	43.82	27.63	90.79	98.32	38.69	86.06	96.32	46.98
Achondroplasia 9	342.25	521.87	215.14	50.53	64.35	36.32	56.06	58.03	41.05	131.45	126.32	60.40	152.38	131.85	47.77
Achondroplasia 10	315.80	614.24	180.01	63.16	47.77	41.05	55.27	56.06	56.06	121.98	127.90	55.66	121.98	127.61	54.48
