# Supplementary material for: Whole-body and segmental analysis of body composition in adult males with achondroplasia using dual X-ray absorptiometry
Source: PLoS One. 2019 Mar 19;14(3):e0213806. doi: 10.1371/journal.pone.0213806 (PMC6424418; doi:10.1371/journal.pone.0213806)
Supplement: S7 Table — (PDF) [file pone.0213806.s007.pdf]

S7 Table: Participant values of fat (%) for each segment.

| | | Trunk | | Right Arm | | | Left Arm | | | Right Leg | | | Left Leg | | |
|---|---|---|---|---|---|---|---|---|---|---|---|---|---|---|---|
| Participant Number | Head & Neck | Thorax | Pelvis | Upper Arm | Fore Arm | Hand | Upper Arm | Fore Arm | Hand | Thigh | Shank | Foot | Thigh | Shank | Foot |
| Control 1 | 21.4 | 20.2 | 24.2 | 24.7 | 14.2 | 23.1 | 23.4 | 15.5 | 22.1 | 21.9 | 27.0 | 18.5 | 23.0 | 15.5 | 20.1 |
| Control 2 | 22.2 | 16.8 | 24.6 | 17.4 | 10.2 | 15.5 | 16.0 | 11.2 | 15.0 | 22.9 | 18.0 | 19.4 | 22.7 | 17.3 | 19.5 |
| Control 3 | 21.8 | 16.9 | 19.1 | 21.5 | 16.7 | 21.0 | 22.5 | 17.2 | 13.0 | 19.8 | 24.1 | 16.9 | 20.7 | 24.3 | 17.3 |
| Control 4 | 22.9 | 33.1 | 36.3 | 31.7 | 19.0 | 28.7 | 31.0 | 20.4 | 27.7 | 31.8 | 32.6 | 27.7 | 32.3 | 30.1 | 27.6 |
| Control 5 | 22.5 | 17.7 | 20.0 | 20.7 | 14.7 | 27.9 | 19.2 | 14.2 | 19.4 | 20.9 | 19.7 | 18.2 | 20.8 | 17.3 | 17.5 |
| Control 6 | 22.2 | 16.3 | 16.8 | 14.6 | 10.4 | 13.2 | 15.7 | 13.9 | 15.6 | 18.0 | 15.2 | 18.8 | 16.8 | 13.9 | 16.0 |
| Control 7 | 22.1 | 16.5 | 17.1 | 16.9 | 12.6 | 16.2 | 17.4 | 13.4 | 20.1 | 17.7 | 16.3 | 14.5 | 18.1 | 16.5 | 14.7 |
| Control 8 | 21.5 | 17.4 | 20.8 | 20.7 | 14.4 | 30.6 | 21.0 | 16.0 | 20.4 | 21.8 | 22.1 | 17.6 | 22.8 | 19.3 | 18.4 |
| Control 9 | 22.2 | 14.2 | 15.0 | 18.4 | 12.3 | 16.3 | 19.1 | 15.2 | 17.9 | 21.2 | 19.2 | 18.0 | 21.7 | 19.9 | 11.9 |
| Control 10 | 23.1 | 26.6 | 26.1 | 37.7 | 25.6 | 24.1 | 35.5 | 22.2 | 29.5 | 30.8 | 31.6 | 25.5 | 32.4 | 28.3 | 25.8 |
| Control 11 | 22.5 | 15.7 | 22.8 | 17.3 | 12.6 | 16.9 | 18.2 | 14.1 | 21.7 | 20.8 | 20.1 | 17.1 | 21.7 | 21.3 | 17.4 |
| Control 12 | 22.4 | 19.4 | 22.4 | 19.0 | 16.5 | 22.1 | 20.0 | 18.5 | 15.8 | 26.1 | 28.2 | 22.3 | 26.4 | 25.9 | 26.5 |
| Control 13 | 22.7 | 18.9 | 23.0 | 20.8 | 14.1 | 19.0 | 19.9 | 14.0 | 23.3 | 22.6 | 23.4 | 27.0 | 24.5 | 22.0 | 19.8 |
| Control 14 | 22.7 | 18.7 | 24.3 | 28.8 | 27.7 | 27.1 | 30.1 | 25.4 | 45.5 | 28.7 | 33.8 | 26.8 | 27.9 | 36.8 | 22.0 |
| Control 15 | 22.1 | 15.7 | 18.5 | 16.8 | 11.7 | 23.7 | 15.4 | 11.5 | 15.9 | 19.3 | 14.7 | 14.8 | 18.7 | 13.0 | 16.7 |
| Control 16 | 22.7 | 25.6 | 26.6 | 22.9 | 18.6 | 20.6 | 22.4 | 15.6 | 18.4 | 27.9 | 32.5 | 23.0 | 28.3 | 33.5 | 24.5 |
| Control 17 | 23.1 | 30.6 | 29.8 | 32.2 | 24.1 | 35.4 | 33.1 | 22.6 | 16.4 | 31.0 | 30.9 | 25.6 | 31.0 | 31.3 | 25.1 |
| Achondroplasia 1 | 23.2 | 35.9 | 34.0 | 44.5 | 31.1 | 29.8 | 47.0 | 31.1 | 40.7 | 35.8 | 40.3 | 31.3 | 35.6 | 35.0 | 29.8 |
| Achondroplasia 2 | 22.8 | 32.0 | 26.9 | 46.9 | 31.0 | 42.6 | 45.0 | 35.0 | 43.4 | 31.1 | 31.8 | 29.4 | 30.0 | 30.9 | 22.8 |
| Achondroplasia 3 | 22.7 | 18.9 | 24.2 | 33.4 | 22.3 | 29.1 | 34.5 | 19.0 | 28.5 | 28.9 | 30.6 | 36.5 | 30.1 | 28.5 | 26.3 |
| Achondroplasia 4 | 22.7 | 26.8 | 28.3 | 37.9 | 27.3 | 31.9 | 39.3 | 27.5 | 34.0 | 33.2 | 35.0 | 30.3 | 33.0 | 34.7 | 27.2 |
| Achondroplasia 5 | 22.5 | 22.6 | 26.6 | 29.5 | 20.8 | 26.2 | 32.6 | 20.5 | 27.3 | 33.5 | 35.1 | 33.5 | 33.0 | 31.1 | 22.1 |
| Achondroplasia 6 | 22.2 | 23.7 | 26.6 | 36.4 | 25.6 | 31.2 | 36.8 | 27.5 | 33.3 | 31.4 | 30.2 | 24.8 | 33.0 | 32.9 | 25.3 |
| Achondroplasia 7 | 22.7 | 30.5 | 32.3 | 37.2 | 24.1 | 32.3 | 38.8 | 23.7 | 34.5 | 34.1 | 32.5 | 29.9 | 34.3 | 32.2 | 23.0 |
| Achondroplasia 8 | 22.6 | 21.7 | 25.2 | 40.2 | 32.8 | 37.1 | 45.6 | 32.8 | 40.6 | 33.9 | 36.7 | 31.8 | 32.4 | 35.6 | 28.4 |
| Achondroplasia 9 | 22.5 | 25.5 | 29.3 | 37.3 | 33.7 | 29.1 | 38.4 | 31.7 | 26.3 | 35.5 | 36.8 | 29.1 | 36.3 | 44.2 | 38.5 |
| Achondroplasia 10 | 22.5 | 27.0 | 27.4 | 34.9 | 24.0 | 29.3 | 34.2 | 25.4 | 31.0 | 33.4 | 39.5 | 30.9 | 31.6 | 39.1 | 28.4 |
