# Supplementary material for: Whole-body and segmental analysis of body composition in adult males with achondroplasia using dual X-ray absorptiometry
Source: PLoS One. 2019 Mar 19;14(3):e0213806. doi: 10.1371/journal.pone.0213806 (PMC6424418; doi:10.1371/journal.pone.0213806)
Supplement: S8 Table — (PDF) [file pone.0213806.s008.pdf]

S8 Table: Participant values of lean mass (kg) for each segment.

Participant Number	Head & Neck	Trunk		Right Arm			Left Arm			Right Leg			Left Leg		
		Thorax	Pelvis	Upper Arm	Fore Arm	Hand	Upper Arm	Fore Arm	Hand	Thigh	Shank	Foot	Thigh	Shank	Foot
Control 1	3.74	19.57	5.90	1.49	0.79	0.26	1.43	0.68	0.23	7.77	1.80	0.59	7.24	1.72	0.58
Control 2	3.88	17.75	4.74	1.85	0.98	0.37	1.96	0.99	0.37	6.68	2.18	0.71	6.52	2.07	0.65
Control 3	3.66	19.46	5.45	1.74	0.97	0.32	1.62	0.88	0.26	7.17	1.94	0.61	6.84	1.85	0.58
Control 4	3.30	13.61	4.41	1.29	0.68	0.21	1.31	0.66	0.22	5.49	1.34	0.41	5.32	1.37	0.39
Control 5	3.73	18.82	5.84	1.87	1.23	0.33	1.97	1.03	0.33	8.45	2.46	0.67	7.92	2.49	0.68
Control 6	3.90	16.28	6.26	1.58	0.90	0.33	1.48	0.83	0.35	6.75	2.13	0.67	6.46	2.02	0.68
Control 7	3.70	17.67	6.43	1.85	0.97	0.38	1.69	0.91	0.34	8.18	2.32	0.80	7.89	2.31	0.79
Control 8	4.10	19.53	6.48	1.74	0.93	0.27	1.79	0.90	0.29	8.38	2.41	0.52	8.21	2.96	0.54
Control 9	3.44	17.00	5.76	1.81	0.96	0.35	1.54	0.95	0.32	6.82	2.14	0.73	6.58	2.09	0.74
Control 10	3.65	19.10	2.35	1.54	0.84	0.27	1.55	0.87	0.29	3.11	2.32	0.71	3.24	2.39	0.68
Control 11	3.70	21.64	6.94	2.37	1.16	0.38	2.18	1.14	0.35	8.98	2.64	0.80	8.79	2.51	0.70
Control 12	3.68	20.86	6.46	2.27	1.08	0.32	2.18	1.06	0.36	7.75	2.21	0.67	7.06	2.16	0.56
Control 13	3.60	19.80	7.37	2.03	1.06	0.35	1.92	1.04	0.34	8.40	2.32	0.66	7.89	2.22	0.65
Control 14	4.54	22.13	7.50	2.31	0.96	0.18	2.09	1.03	0.24	9.45	2.52	0.70	9.41	2.18	0.67
Control 15	3.50	21.45	5.98	2.03	1.09	0.32	1.97	1.02	0.33	7.65	2.11	0.70	7.50	2.20	0.62
Control 16	3.43	23.41	7.40	2.48	1.05	0.34	1.42	1.05	0.36	9.22	2.14	0.60	8.88	2.05	0.59
Control 17	3.82	22.18	7.60	2.01	1.02	0.28	2.00	1.05	0.36	9.48	2.40	0.55	9.51	2.41	0.64
Achondroplasia 1	4.22	16.87	5.12	0.99	0.64	0.24	0.95	0.66	0.18	5.69	1.33	0.48	5.77	1.50	0.46
Achondroplasia 2	3.51	15.32	4.53	0.72	0.53	0.18	0.63	0.46	0.17	4.42	1.40	0.47	4.27	1.27	0.51
Achondroplasia 3	3.81	15.44	5.55	0.83	0.49	0.20	0.77	0.60	0.18	4.42	1.00	0.40	4.20	1.01	0.41
Achondroplasia 4	3.49	14.62	4.76	0.83	0.51	0.20	0.71	0.52	0.19	3.89	1.13	0.39	4.05	1.10	0.41
Achondroplasia 5	4.32	16.17	5.06	1.10	0.62	0.25	0.79	0.63	0.28	3.87	1.16	0.42	3.74	1.13	0.54
Achondroplasia 6	4.15	18.17	6.58	0.94	0.60	0.24	0.78	0.61	0.21	2.26	1.30	0.49	4.82	1.24	0.51
Achondroplasia 7	3.64	14.80	5.05	0.87	0.52	0.20	0.72	0.51	0.19	4.64	1.34	0.42	4.59	1.32	0.47
Achondroplasia 8	3.04	14.39	3.99	0.69	0.41	0.19	0.53	0.53	0.17	3.75	1.08	0.36	3.62	1.07	0.39
Achondroplasia 9	4.51	17.83	5.95	0.93	0.59	0.24	0.85	0.57	0.29	5.59	1.54	0.55	5.26	1.34	0.42
Achondroplasia 10	4.46	20.25	6.78	1.38	0.80	0.27	1.22	0.73	0.28	5.01	1.41	0.49	5.07	1.36	0.52
